# Intramedullary Nailing *Versus* Minimally Invasive Plate Osteosynthesis for Distal Tibial Fractures: A Systematic Review and Meta‐Analysis

**DOI:** 10.1111/os.12575

**Published:** 2019-12-10

**Authors:** Xiao‐kai Liu, Wen‐nan Xu, Qing‐yun Xue, Qing‐wei Liang

**Affiliations:** ^1^ Joint Surgery Department Chaoyang Central Hospital Chaoyang City Liaoning Province China; ^2^ Department of Orthopaedic Beijing Hospital, National Center of Gerontology Beijing China; ^3^ Department of Orthopaedic The First Affiliated Hospital of China Medical University Shenyang Liaoning Province China

**Keywords:** tibia fracture, nailing, plate

## Abstract

To evaluate the application, safety and efficacy of the patients treated with intramedullary nailing (IMN) and minimally invasive plate osteosynthesis (MIPO) in distal tibia fractures. Following the Preferred Reporting Items for Systematic Reviews and Meta Analysis (PRISMA) guidelines, we searched databases PubMed, Cochrane library, EMBASE and Web of Science from inception of the database up to 10 October 2018, using the keywords “distal tibia fractures”, “plate”, “intramedullary nailing” and “RCT” to identify randomized clinical trials about distal tibia fractures. The included studies were assessed by two researchers according to the Cochrane risk‐of‐bias criteria. The primary outcome of measurement included operation time, malunion rate, nonunion/delayed union rate, and wound complication. Data analysis was conducted with Review Manager 5.3 software. A total of 10 RCTs involving 911 patients fulfilled the inclusion criteria with 455 patients in the IMN group and 456 patients in the MIPO group. There were no significant differences in radiation time, nonunion or delayed union rate, union time and operation time between the two groups. Patients treated with MIPO had lower incidence of malunion compared with IMN (RR = 1.85, 95%CI: 1.21 to 2.83, *P* = 1.00), while IMN seemed to have lower surgical incision complications whether in closed or opening fractures (RR = 0.49, 95%CI 0.33 to 0.73, *P* = 0.43). But in patients classified as 43A, the result of subgroup analysis suggested that there was no significant inwound complication between the two groups. MIPO was superior in preventing malunion compared with IMN, and intramedullary nailing appeared to have lower wound complications. However, in patients with 43A distal tibial fractures, MIPO was more recommended for its prevention of malunion. No matter which method we choose, we should notice and prevent the associated complications.

## Introduction

Distal tibia fractures occurred in patients commonly as a result of a force directed from the foot towards the leg in high energy traumatic events, such as falling down, traffic accident, motorcycle accident, or sport injury^1^. Considering the need for anatomical reduction and rigid internal fixation sometimes, it might be necessary to widely expose the surrounding tissues of the fracture, which could cause delayed union or nonunion owing to over‐destruction of soft tissue and blood supply around the fracture^2^. The common surgical procedures included intramedullary nailing, minimally invasive plate osteosynthesis (MIPO), open reduction and internal fixation (ORIF), and external fixation.

In recent years, intramedullary nails were widely used because of their successful outcomes and minimal damage to bone and soft tissue, especially in open fractures^3^. Some researchers reported that intramedullary nailing was an effective technique for stabilizing distal tibia^4^, ^5^. However, delayed bone healing, reoperation, and a high incidence of primary and secondary malalignment have also been reported, especially in distal and proximal tibial fractures, which may associated with the large cavity^6^.

The treatment of distal tibial fractures with plate provided a reliable fixation by achieving anatomical reduction and restoring alignment of the limb, which could allow early rehabilitation exercise for patients. But the high incidence of wound problems and reoperation was also reported^7^. With the development of minimally invasive technology, minimally invasive plate osteosynthesis (MIPO) has become an excellent method^8^. It protected the subcutaneous soft tissue of anterior medial tibia and enabled adequate soft tissue coverage overlying the plate with less wound complications^9^, ^10^.

Obviously, as two ideal minimally invasive methods, intramedullary nailing (IMN) and MIPO have their own advantages and disadvantages in the treatment of distal tibial fractures. But the best treatment for distal tibial fractures remains controversial, currently. The aim of our study was to evaluate randomized controlled trials, which compared the effect of intramedullary nailing and MIPO in distal tibial fractures.

## Materials and Methods

### *Search Strategy*

We conducted this study according to the Preferred Reporting Items for Systematic Reviews and Meta‐Analysis (PRISMA) statement^11^. The research protocol for this review was determined by all authors before the literature searches were begun. The electronic databases including MEDLINE, EMBASE, Web of Science, and Cochrane Library were searched for relevant studies published from inception of database to 10 October 2018 with no language restrictions. We used search strategy of (((random*[Title/Abstract] OR prospect*[Title/Abstract] OR RCT*[Title/Abstract]))) AND (((((“Fracture Fixation, Intramedullary”[Mesh]) OR ((intramedul*[Title/Abstract] OR nail*[Title/Abstract])))) AND ((plate*[Title/Abstract] OR MIPO[Title/Abstract]))) AND ((“Tibial Fractures”[Mesh]) OR ((fracture*[Title/Abstract]) AND ((“distal tibia”[Title/Abstract] OR “distal tibial”[Title/Abstract]))))) to identify randomized clinical trials about distal tibia fractures.

### *Inclusion and Exclusion Criteria*

Articles were assessed independently by two researchers using pre‐designed eligibility forms according to the eligibility criteria, defined prospectively. Any disagreement between investigators was resolved by consensus. We also examined the reference lists of each comparative study and review to identify additional relevant studies.

Trials were selected based on the following inclusion criteria: (i) people: patients with distal tibial fractures (extra‐articular fracture); (ii) intervention: IMN; (iii) comparison: MIPO; (iv) outcome measures: reported at least one of the following outcomes: malunion, delay union or nonunion, union time, wound complications, radiation time and operation time; and (v) study design: randomized controlled trials (RCTs).

Exclusion criteria included: (i) intra‐articularly tibial fractures were involved; (ii) neither of the outcomes was available; (iii) no control data was provided; and (iv) only open reduction and internal fixation with plate.

### *Data Extraction*

Data which contained basic information and major outcomes were independently and carefully extracted from the included studies into a standardized Excel file by two researchers and checked by a third investigator. All differences and disagreements between the two evaluators were settled through discussion and consensus. The recorded items of studies included author name, publication year, country, description of distal tibial fracture, mean age, BMI, gender, fibular fixation, follow‐up, wound type and AO/OTA classification. The primary outcomes were operation time, malunion rate, nonunion/delayed union rate, and wound complication. Secondary outcomes included radiation time and union time.

### *Quality Assessment*

The methodological quality for the included studies was assessed independently by two researchers based on Cochrane risk‐of‐bias criteria and each quality item was graded as low risk, high risk, or unclear risk. The seven items used to evaluate bias in each trial included: the randomization sequence generation, allocation concealment, blinding of participants and personnel, blinding of outcome assessment, incomplete outcome data, selective reporting, and other bias. These studies were independently assessed by two authors and any controversy was resolved by a final consensus.

### *Statistical Analysis*

Data from the included studies were analyzed with Review Manager 5.3 software. Dichotomous variables (malunion, nonunion/delayed union, and complication) were expressed as risk ratio (RR) and 95% confidence interval (CI), while mean difference (MD) or the standardized mean difference (SMD) was calculated in continuous data (operative time, union time, and radiation time). If median, the size of a sample, mean, ranges or 95%CI of the continuous variable reported in the study, mean value and standard deviation are calculated using the method described by Hozo^12^ and the calculator of Review Manager 5.3 software. Q and *I* ^*2*^ test were used to estimate the heterogeneity among studies. When *I* ^*2*^ were less than 50% or *P* > 0.1, a fixed‐effect model was applied for the meta‐analysis, when on the contrary, a random‐effect model should be adopted. Sensitivity analysis was performed to evaluate the stability of the results (when necessary), and subgroup analysis was conducted according to AO/OTA, patient age and wound type. Forest plots were used to present the results of the individual studies and respective pooled estimates of effect size.

## Results

### *Study Selection*

**Figure 1:**
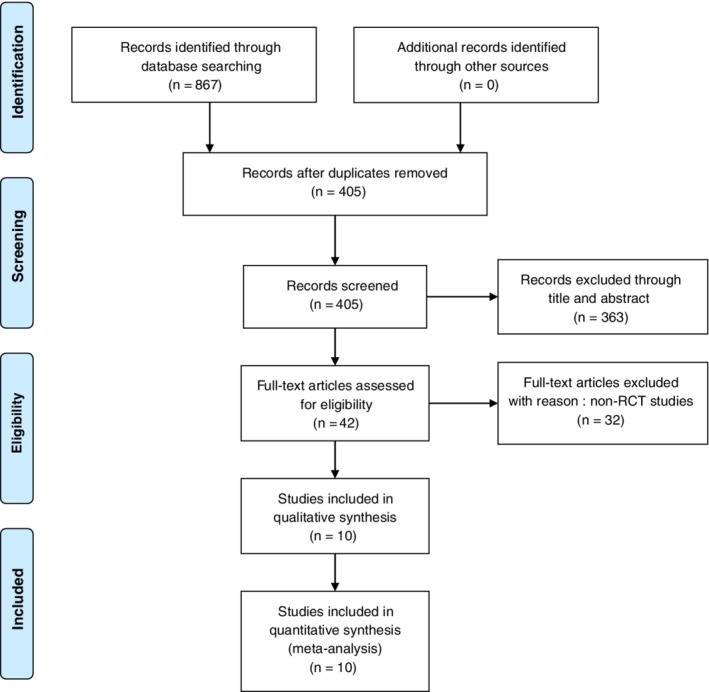
Flow chart of inclusion and exclusion for included studies.

A total of 867 potentially relevant citations were extracted from the four electronic databases. After removing the duplicates and reading the abstract and title, 42 studies were screened for relevance. Eventually, 10 RCTs with 911 patients were considered to meet the eligibility criteria and included in the systematic review after screening the full‐text. All the studies were published between 2005 and 2018 and the process of selecting appropriate studies is shown in a flow diagram (Fig. 1).

### *Characteristics of Included Studies*

A total of 911 patients with distal tibial fractures were included in the meta‐analysis, with 455 patients who underwent intramedullary nailing and 456 patients who were treated with MIPO. All of the included studies reported the content about author, publication year, country, type of treatment, mean age, follow‐up, wound type, and AO/OTA classification. Most of the eligible studies were conducted from Asia, and only three studies were from non‐Asian countries. The sample size ranged from 24 to 321 with 599 male patients and 312 female patients, and the average age ranged from 34 to 53 years. The average follow‐up time for most studies was 12 months, the longest average follow‐up time was 24 months, and the shortest was 3 months. Only two studies provided data about BMI, and five studies have reported the reaming of intramedullary nailing. There was no report on the fixation of combined fibula fracture in three studies. In addition, the fractures classified by AO/OTA system and Gustilo classification were listed in Table 1 together with type of internal fixation (Table 1).

**Table 1:** The characteristics of included studies

Author	Year	Type	Mean age	Gender(male/female)	Fibular fixation	Follow‐up(months)	Wound type	AO/OTA classification
Costa *et al*.^23^	2018	IMN(NR)/MIPO	46.3 ± 16.3/45.8 ± 16.3	IMN:96/65; MIPO:101/59	IMN/plate:10/12	12	Closed	42A、42B、42C、43A
Daolagupu *et al*.^16^	2017	IMN(NR)/MIPO	35.19/39.09	IMN:17/4; MIPO:15/6	IMN/plate:8/14	12	Closed	43A1,A2,A3
Wani *et al*.^19^	2017	IMN(NR)/MIPO	36.4 ± 9.7/38.4 ± 8.7	IMN:22/8; MIPO:20/10	IMN/plate:6/8	12	Closed	42A1,42A2,42A3
Fang *et al*.^17^	2016	IMN(unreaming)/MIPO	35/38.6	IMN:19/9; MIPO:21/7	IMN/plate:20/19	29.4/26.3	Closed,type I,II	42A,B,C
Polat *et al*.^22^	2015	IMN(reaming)/MIPO	34/36.4	IMN:9/1; MIPO:7/8	IMN/plate:2/4	23.8/22.7	Closed	42A1,A2,A3
Chen *et al*.^13^	2014	IMN(reaming)/MIPO/OP(open reduction)	IMN:53.0 ± 8.1MIPO:40.8 ± 7.3OP:47.0 ± 9.0	IMN:35/25; MIPO:33/27; OP:31/29	NR	12	Closed	42A,B
Li *et al*.^14^	2014	IMN(reaming)/MIPO	44/43	IMN:41/5; MIPO:38/8	NR	14.6/15.2	Closed,type I,II	42A,B,C
Mauffrey *et al*.^21^	2012	IMN(NR)/Lock‐plate(MIPO)	50/33	IMN:7/5; MIPO:9/3	IMN/plate:2/0	12	Closed,type I	43A
Guo *et al*.^18^	2010	IMN(reaming)/MIPO	44.2/44.4	IMN:26/18; MIPO:24/17	IMN/plate:0/0	12	Closed	43A1,A2,A3
Muhammad *et al*.^43^	2016	IMN/MIPO	38.79 ± 10.47/39.63 ± 11.73	IMN:29/14; MIPO:30/13	NR	3	Closed	43A

IMN, intramedullary nailing, MIPO, minimally invasive plate osteosynthesis; NR, not reported.

### *Quality Assessment of the Eligible Studies*

**Figure 2:**
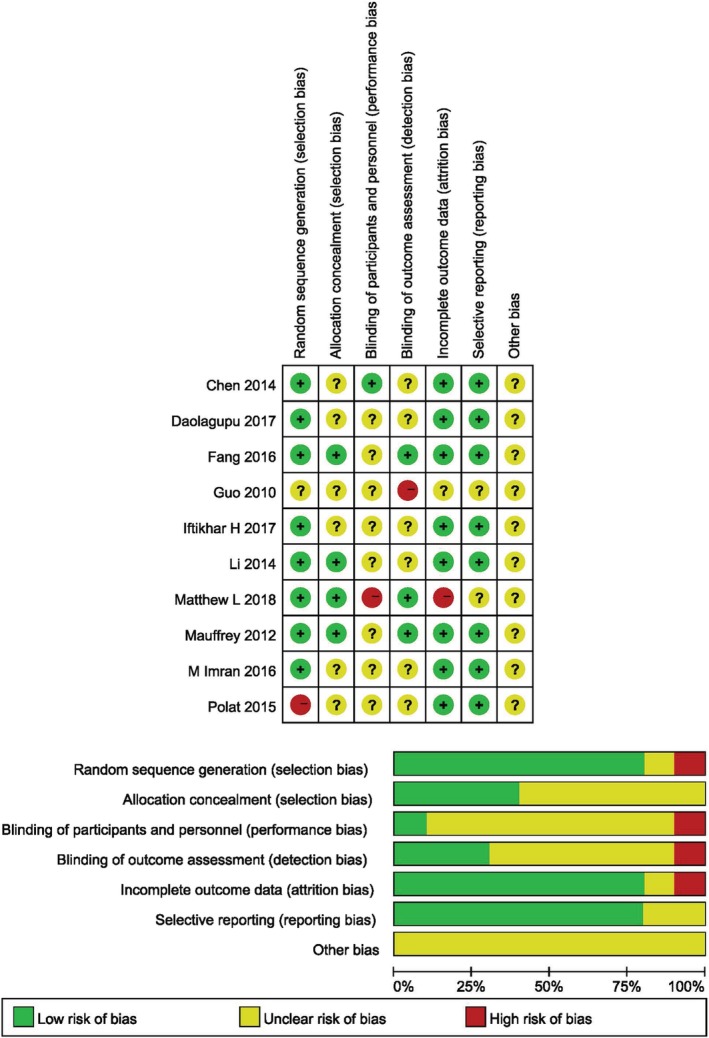
Risk of bias graph and Risk of bias summary.

All of the RCTs reported the detailed data about fibular fixation except two studies^13^, ^14^. Ten RCTs^13^, ^14^, ^15^, ^16^, ^17^, ^18^, ^19^, ^20^, ^21^, ^22^ were assessed by the Cochrane Handbook, and the detailed information of studies was displayed in Fig. 2. Two studies^14^, ^17^ mentioned that the randomization was realized by a computer‐assisted tool, and allocation concealment was performed by opaque envelope. The studies conducted by Mauffrey *et al*.^21^, Daolagupu *et al*.^16^, and Costa *et al*.^23^ were also randomized by computer; among them, Costa *et al*. used a secure, centralized, web‐based randomization service *via* a minimization algorithm provided by an accredited clinical trials unit. They also pointed out that, because of visible surgical scars, patients can easily identify the type of fracture fixation, the patients and surgeons could not be blinded to their treatment. Patients in the study by Polat *et al*. were randomized by flipping a coin without reporting allocation concealment and blind. Allocation concealment of five studies^13^, ^16^, ^18^, ^19^, ^22^ were considered at unclear risk of bias. Guo *et al*.^18^ did not provide the detailed the method of randomization and sufficient data about the loss of patients. Blind method was used for both the patient and the operator in the study of Chen, and the blind was exposed on the day of operation (Fig. 2).

### *Operative Time*

**Figure 3:**
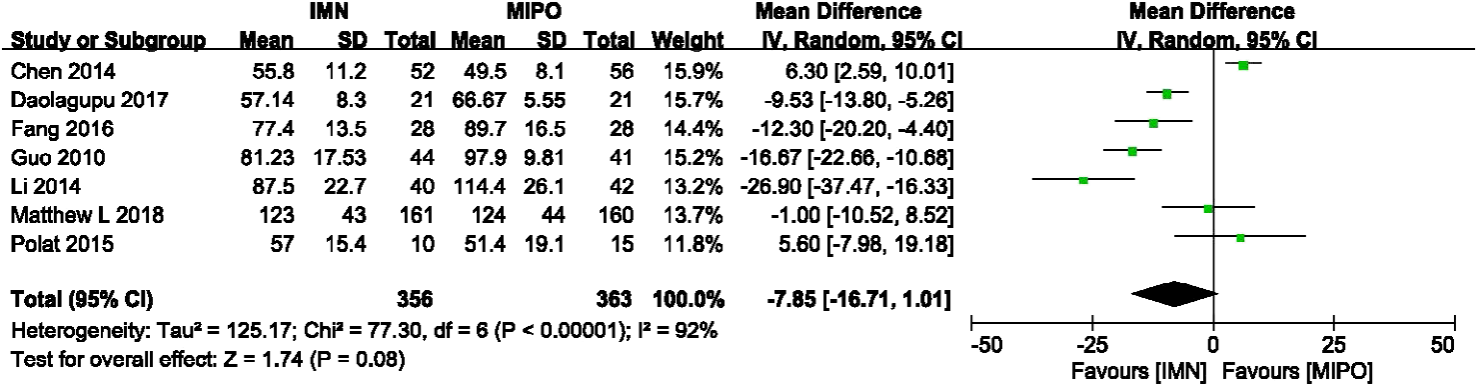
Comparison of operative time between IMN and MIPO. The pooled result showed that IMN did not provided less operative time compared with MIPO.

Seven RCTs, including 719 patients, reported the operative time. Figure 3 showed the result of meta‐analysis comparing operative time in both groups with random‐effect model (*I* ^*2*^ = 92%, *P* < 0.00001). There was no significant difference between IMN group and MIPO group (*MD* = −7.85, 95%*CI*: −16.71 to 1.01, *P* = 0.08). Intramedullary nail did not show less surgery time compared with MIPO. While there is decreased heterogeneity among the studies in the subgroup, analysis indicated that age and AO/OTA classification may have an effect on this outcome: patients whose age is less than 40 years and classified as 43A appear to have less operative time in IMN (Fig. 3) (Table 2).

**Table 2:** Subgroup analysis of operative time between IMN and MIPO (based on AO/OTA, age and wound type)

Variable	No. of Trials	No. of Participants	Mean Difference	*P* value
AO/OTA				
43A	2	127	−12.78 [−19.75, −5.81]	0.0003
42	4	271	−6.75 [−22.37, 8.87]	0.4
Age(years)				
>40	4	596	−9.25 [−24.62, 6.12]	0.24
<40	3	123	−7.58 [−14.89, −0.26]	0.04
Wound type				
Closed and open fracture	2	138	−19.16 [−33.44, −4.88]	0.09
Only closed fracture	5	581	−3.50 [−13.42, 6.41]	0.49

*P* value <0.05 means there is a statistical difference in the results.

### *Radiation Time*

**Figure 4:**
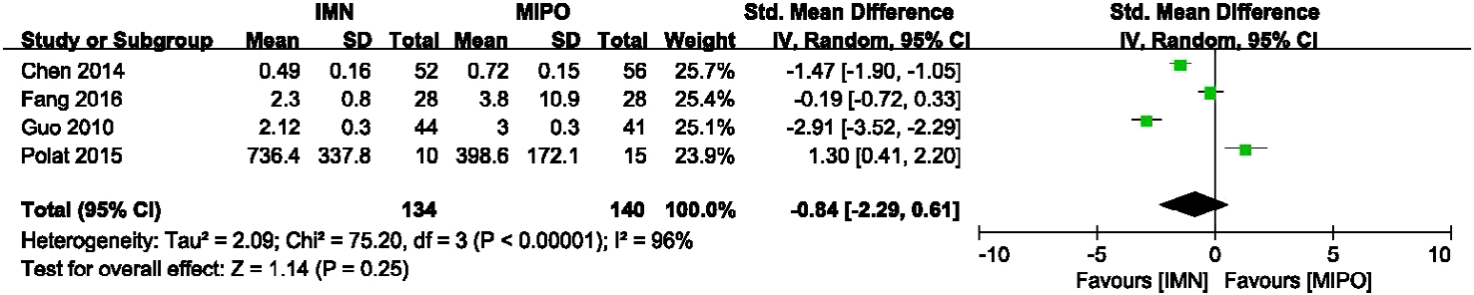
Comparison of radiation time between IMN and MIPO. The result showed no statistical difference in radiation time between two groups.

Four studies containing 274 patients compared the radiation time in both group. In the study of Guo *et al*.,^18^ the 95% confidence interval, mean, and sample size are given without standard deviation. We calculated the standard deviation by the calculator of Review Manager software. Although the calculated mean is different from the actual reported mean in this study, the results of meta analysis showed that there was no significant difference between the IMN and MIPO whether calculated by actual data or calculated data. Figure 4 reported the result of radiation time with random‐effect model (*I* ^*2*^ = 96%, *P* < 0.00001). There was no significant difference in IMN group compared with MIPO group (*SMD* = ‐0.84, 95%*CI*: −2.29 to 0.61, *P* = 0.25) (Fig. 4).

### *Union Time*

**Figure 5:**
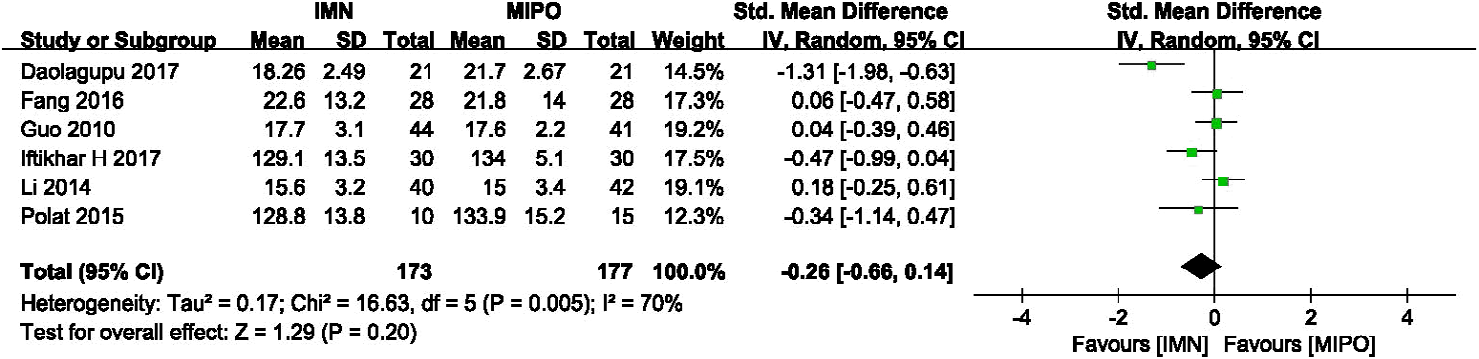
Comparison of union time between IMN and MIPO. The pooled data indicated that there was no significant difference in union time between IMN and MIPO.

Union time was reported in six studies with 350 patients. The pooled data was analyzed by random‐effect model (*I* ^*2*^ = 70%, *P* = 0.005) and there was no significant difference between IMN group and MIPO group. Figure 5 showed the result of meta‐analysis between both groups (*SMD* = −0.26, 95%*CI*: −0.66 to 0.14, *P* = 0.20). The subgroup analysis also suggested, whether in closed or opening fracture, no significant difference in union time was found between two groups, and AO/OTA classification of the fracture (43A/42) was not associated with union time in distal tibial fractures (Fig. 5) (Table 3).

**Table 3:** Subgroup analysis of union time between IMN and MIPO (based on AO/OTA, age and wound type)

Variable	No. of Trials	No. of participants	Std. mean difference	*P* value
AO/OTA				
43A	2	127	−0.61 [−1.93, 0.71]	0.36
42	4	223	−0.10 [−0.42, 0.23]	0.55
Age(year)				
>40	2	167	0.11 [−0.20, 0.41]	0.49
<40	4	183	−0.50 [−1.06, 0.06]	0.08
Wound type				
Closed and open fracture	2	138	0.13 [−0.20, 0.46]	0.44
Only closed fracture	4	212	−0.49 [−1.05, 0.07]	0.09

*P* value <0.05 means there is a statistical difference in the results.

### *Malunion*

**Figure 6:**
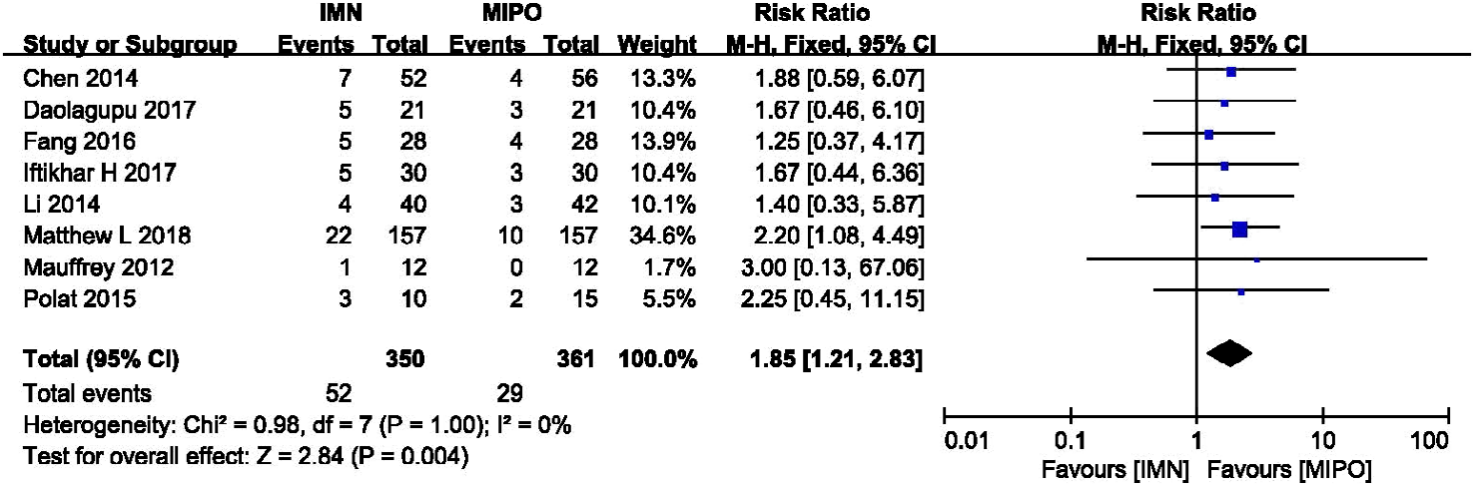
Comparison of malunion between IMN and MIPO. The outcome suggested that IMN was more inclined to have malunion than MIPO.

Eight studies involving 711 patients reported incidence of malunion. There was significant difference between IMN group and MIPO group according to the meta‐analysis with fixed‐effect model (*I* ^*2*^ = 0, *P* = 1.00). Figure 6 listed the result of malunion in both groups (*RR* = 1.85, 95%*CI*: 1.21 to 2.83, *P* = 0.004), and more incidence of malunion was showed in IMN compared with MIPO, especially in patients with closed fracture and over the age of 40 (Fig. 6) (Table 4).

**Table 4:** Subgroup analysis of malunion between IMN and MIPO (based on AO/OTA, age and wound type)

Variable	No. of trials	No. of participants	RR (risk ratio)	*P* value
AO/OTA				
43A	2	66	1.86 [0.56, 6.13]	0.31
42	5	331	−6.75 [−22.37, 8.87]	0.11
Age(years)				
>40	4	528	2.02 [1.16, 3.50]	0.01
<40	4	183	1.60 [0.82, 3.12]	0.17
Wound type				
Closed and open fracture	3	162	1.43 [0.59, 3.44]	0.43
Only closed fracture	5	549	2.00 [1.23, 3.25]	0.005

*P* value <0.05 means there is a statistical difference in the results.

### *Nonunion or Delayed Union*

**Figure 7:**
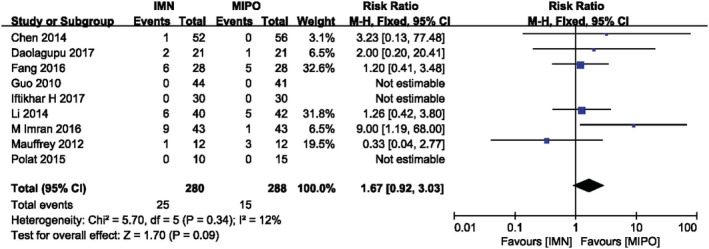
Comparison of nonunion or delayed union between IMN and MIPO. The pooled result indicated a nonsignificant difference in nonunion and delayed union rate.

Nonunion or delayed union were serious postoperative complications. Nine RCTs involving 568 patients compared these problems between IMN and MIPO with fixed‐effect model (*I* ^*2*^ = 12, *P* = 0.34). There was no significant difference between two groups (*RR* = 1.67, 95%*CI*: 0.92 to 3.03, *P* = 0.09). But patients with closed distal tibial fracture might have an increased incidence of nonunion or delayed union in IMN group (Fig. 7) (Table 5).

**Table 5:** Subgroup analysis of nonunion/delayed union between IMN and MIPO (based on AO/OTA, age and wound type)

Variable	No. of trials	No. of participants	RR (risk ratio)	*P* value
AO/OTA				
43A	4	237	2.40 [0.88, 6.58]	0.09
42	5	331	1.32 [0.63, 2.78]	0.46
Age(years)				
>40	4	299	1.04 [0.43, 2.53]	0.93
<40	5	269	2.43 [1.06, 5.56]	0.04
Wound type				
Closed and open fracture	3	162	1.02 [0.50, 2.07]	0.95
Only closed fracture	6	406	5.06 [1.34, 19.14]	0.02

*P* value <0.05 means there is a statistical difference in the results.

### *Wound Complication*

**Figure 8:**
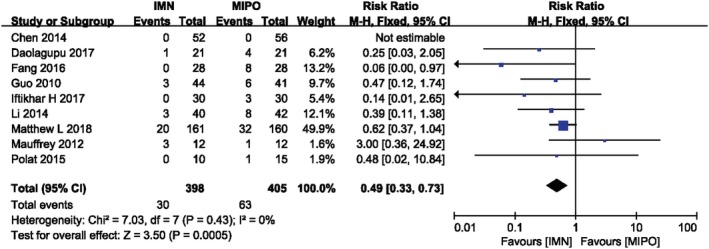
Comparison of wound complication between two groups and IMN had lower incidence rate in wound complication than MIPO.

The wound problems was analyzed in nine studies containing 803 patients. We analyzed wound problems, such as superficial, deep wound infection and delayed wound healing etc. Wound complication was discovered in 30 of 398 patients treated by intramedullary nail, and in 63 of 405 patients treated by MIPO. There was no heterogeneity among these studies (*I* ^*2*^ = 0%, *P* = 0.43). Figure 8 showed the result of meta‐analysis with fixed‐effects model which indicated the IMN group had significantly lower incidence of wound complication than the MIPO group (*RR* = 0.49, 95%*CI* 0.33 to 0.73, *P* = 0.0005), whether in closed fractures or opening fractures. Although this result without heterogeneity suggested that IMN has lower surgical incision complications than MIPO, patients whose fractures classified as 43A seemed to be more inclined to have no statistical difference between the two groups, and further studies and strict grouping were still needed on the differences of postoperative incision complications between the two surgical methods (Fig. 8) (Table 6).

**Table 6:** Subgroup analysis of wound complication between IMN and MIPO (based on AO/OTA, age and wound type)

Variable	No. of trials	No. of participants	RR (risk ratio)	*P* value
AO/OTA				
43A	3	151	0.61 [0.25, 1.51]	0.29
42	5	331	0.22 [0.08, 0.59]	0.002
Age(year)				
>40	5	620	0.61 [0.40, 0.94]	0.03
<40	4	183	0.15 [0.04, 0.54]	0.004
Wound type				
Closed and open fracture	3	162	0.38 [0.16, 0.90]	0.03
Only closed fracture	5	641	0.53 [0.34, 0.83]	0.006

*P* value <0.05 means there is a statistical difference in the results.

### *Sensitivity Analysis*

We analyzed the sensitivity analysis of operative time, radiation time, and union time because of its high heterogeneity in meta‐analysis. The results of the union time after excluding the studies conducted by Daolagupu *et al*.^16^ was quite different from that of the total studies, and the heterogeneity reduced from *I* ^*2*^ = 70% to 11%. But the other two variables, operative time and radiation time, excluding the results of the other studies, did not find such a slight difference. In addition, we also used different effect models to estimate the stability of the results. The sensitivity analysis of malunion, nonunion/delayed union, and wound problems were consistent whether using fixed‐effects model or random‐effects model.

## Discussion

The purpose of this study was to investigate which minimally invasive treatment was more suitable for distal tibial fractures by comparing IMN and MIPO in 10 studies that included RCTs. We found that there were no significant differences in radiation time, nonunion or delayed union rate, union time and operation time between the two groups. But decreased heterogeneity in the subgroup indicated that age and AO/OTA classification might have an effect on operation time between the two groups. Moreover, in the subgroup analysis of union time, the result suggested whether AO/OTA classification (43A/42) or type of fracture (closed or opening fracture) was not associated with union time in two groups. Patients treated with MIPO had lower incidence of malunion, while IMN seemed to have lower surgical incision complications whether in closed or opening fractures. But in patients classified as 43A, the result of subgroup analysis suggested that there was no significant difference in wound complication between the two groups.

Our study suggested that patients who underwent MIPO were more superior in malunion, and IMN did not have advantages in radiation time, nonunion or delayed union rate, union time and operation time compared with MIPO. Whether in closed or opening fractures, IMN appeared to have lower incidence of wound complications, but MIPO was recommended for the treatment of distal tibial fractures classified as 43A for its advantage in preventing malunion.

Although each study included distal tibial fractures, they had slightly different criteria in definition of distal tibial fractures. There were two types of description about distal tibia fracture found in all of the studies. One was the extent of distal tibial fracture within two “Müller squares” of the ankle joint or the distal third part of the tibia, and the regions described by those two were basically the same. Another description about distal tibial fracture was located between 3 cm and 12 cm from the tibial plafond. Whatever the classification method was, all distal tibial fractures included in the study were extra‐articular fractures.

Considering slightly different criteria for the definition of distal tibial fractures in the included studies, there were different definitions associated with different AO/OTA classification of distal tibial fractures. Subgroup analysis based on AO/OTA classification was recommended and reasonable, and we classified them as 43A and 42 according to the included studies.

Malunion was defined as varus/valgus deformity >5° in the coronal plane, anterior/posterior angulation >10° in the sagittal plane, a rotational deformity >10°, and shortening >10 mm^19^. When analyzed, the incidence of malunion, although part of RCTs in our meta‐analysis did not report significant difference between two groups, the pooled data pointed a higher incidence of malunion in IMN group than in MIPO group. Costa *et al*.^23^ conducted a multicenter randomized trial including 321 patients with an acute, displaced, extra‐articular distal tibia fracture from April 2013 to April 2016. They allocated patients into IMN group (n = 161) and MIPO group (n = 160) with a 12 months follow‐up. Considering the higher missing rate at 12‐month postoperation, we only extracted the data about malunion at 6‐week postoperation with the maximum missing rate of 13%. Costa *et al*.^20^, ^23^ suggested there was no significant difference about lateral deformity (*P* = 1.000) and anteroposterior deformity (*P* = 0.081) between IMN group and plate group; nevertheless they found shortening deformity (>10 mm) was associated with IMN group (*P* = 0.028). Moreover, Wani *et al*.^19^ reported that patients treated with IMN had significantly higher rotational malalignment than plate, but they did not find any significant difference in varus or valgus deformity and anterior/posterior angulation.

With the development of IMN design and adjunctively surgical techniques, surgeons began to apply these methods, like angle‐stable and multi‐directional distal screw or blocking screw in distal tibia fractures to maintain reduction, alignment and prevent malunion^24^, ^25^. A multi‐center pilot study conducted by Höntzsch^26^ showed that this technique help improve the stability in axial and torsional loading, which is statically and dynamically higher than conventional IMN. A retrospective study performed by van Maele *et al*.^27^ included 184 distal tibial fractures associated with fibula fracture. The result reported a clear benefit of angular‐stable locking system (ASLS) about increasing the stability of IMN by measured coronal and sagittal alignment 3–6 months after IMN. Moongilpatti Sengodan *et al*.^28^ performed a prospective study of approximately 20 patients with distal tibial metaphyseal fractures, and all of the participants were treated with statically locked intramedullary nailing with supplementary blocking screw. They suggested that to supplement with blocking screw could achieve and maintain the reduction of distal tibialmetaphyseal fractures, which could extend the indication of intramedullary nailing. The similar result had been reported in Shahulhameed *et al*.^29^ with a new technique for precise placement of poller screws in metaphyseal fractures of tibia.

In terms of delayed union or nonunion rate, we found that the pooled data of meta‐analysis did not show any significant difference in both groups. Seven studies reported both union time and incidence of nonunion/delayed union. The incidence of nonunion and delayed union was within the scope of the rates reported previously in the literature^17^.

With the exception of Chen *et al*.^13^ and Li *et al*.,^14^ all of the RCTs reported the detailed data about fibular fixation. Mohammad Javdan *et al*.^30^ conducted a RCT study about the role of fibular fixation in the distal tibial fracture(AO/OTA 43 A1‐3) combined with fibular fracture, which included 24 and 25 patients in the case and control group. IMN and plate were used in both groups, and patients without fibular fixation was control group. They did not observe any significant difference between the two groups in malunion, union time, and complication. They concluded that fibular fixation in the treatment of tibia distal fractures provides no improvement. A study conducted by Taylor *et al*.^31^ recruited 98 patients with concurrent non‐comminuted distal third tibia fracture and fibula fracture who underwent IMN of distal tibia fractures with or without fibular fixation. They also found no statistically significant difference between the fibular fixation group and the non‐fixation group in operative time, malalignment, union rate, delayed union rate, and wound complication. Nevertheless, Egol *et al*.^32^ separated distal tibia‐fibula fracture into two groups, with IMN fixation of tibia and plate fixation of fibula fracture or without fibula fixation in a retrospective study. They suggest that fibula fixation with plate was significantly associated with maintenance of reduction beyond 12 weeks (odds ratio = 0.03; *P* = 0.036). Therefore, we believed that patients with distal tibial fractures combined with fibula fractures, and whether the fibula fractures need to be fixed, required further RCT studies.

Common wound complications included superficial infection, deep infection, erythema, purulent drainage, dehiscence and persistent serous drainage etc. The incidence of wound complication in IMN group was lower than that in the MIPO group in almost all of the RCTs we included, except the study conducted by Mauffrey *et al*.^21^. Pooled data in the meta‐analysis indicated that distal tibia fractures treated with intramedullary nailing was associated with lower incidence of wound complication, but there was no significant difference in patients with 43A between the two groups.

Some researchers reported that the incidence of infection was higher in plate fixation with open reduction compared to intramedullary nailing^33^. In a meta‐analysis conducted by Kwok *et al*.^34^, they did not find any significant difference about the incidence of infection in both plate and intramedullary nailing group. We found that they analyzed the data extracted from retrospective cohort studies and prospective randomized studies, in addition, patients allocated in experimental group (IMN/MIPO) and control group (IMN/ORIF) were not performed subgroup analysis.

Patients who experienced deep infections were always subsequently treated with debridement, remove of device and antibiotics, while for superficial infections, they were often resolved by debridement and oral antibiotics^35^.

The wound complications were related to the surgical techniques and other factors, such as patient co‐morbidities, conditions and contamination of skin and soft tissue, surgery time, mechanisms of injury and open fractures etc.; all of these play an important role in wound complications^36^.

Although our results showed that IMN has an advantage in wound complications, the incidence of malunion in IMN was higher than that of MIPO, and it was not suitable for patients with intra‐articular fractures, narrow medullary canal, periprosthetic fractures, and malunion^37^.

Compared with previous meta‐analysis^34^, ^38^, ^39^, ^40^, ^41^, all of the studies included IMN and plate (ORIF/MIPO), but only Xue *et al*.^39^ performed the subgroup‐analysis about MIPO. In addition, most of the included studies were retrospective, nonrandomized trials. We also found that although the latest meta‐analysis conducted by Guo *et al*.^42^ searched from inception of database to August 2017 with 482 patients, there was no subgroup analysis, sensitivity analysis, and publication bias for the outcomes with high heterogeneity. In addition, they also did not perform the subgroup analysis of MIPO. Therefore, we re‐researched related RCTs from the inception time of the four databases to 10 October 2018. We added new RCT studies, and the total number of patients included in the meta‐analysis increased to 889 from the previous 482. Considering the heterogeneity of some results, we performed subgroup analysis and sensitivity analysis, trying to explain the source of its heterogeneity.

There were some limitations in this study. First, only four RCT studies included provided radiation time, which may be one of the reasons for the high heterogeneity of the result, meaning it was not suitable to perform subgroup analysis. Second, only a few studies reported the result of knee pain and foot/ankle function score, which made them unsuitable to perform meta‐analysis, and further studies were needed.

### *Conclusion*

Both intramedullary nailing and MIPO are ideal minimally invasive methods for the treatment of distal tibial fractures. MIPO is superior to IMN in preventing malunion, and intramedullary nailing is inclined into reducing wound complications. However, in patients whose distal tibial fractures are 43A, there is no statistical difference between the two methods in wound complications, and MIPO is recommended for these patients.

### *Authorship declaration*

All authors listed meet the authorship criteria according to the latest guidelines of the International Committee of Medical Journal Editors, and that all authors are in agreement with the manuscript.
